# The valency of consumers’ perceptions toward cultured meat: A review

**DOI:** 10.1016/j.heliyon.2024.e27649

**Published:** 2024-03-09

**Authors:** C.Z. Tsvakirai

**Affiliations:** The University of South Africa, School of Business Leadership, C/O Jadanel and Alexandra Ave, Midrand, 1686, South Africa

## Abstract

The prospects of developing a global market for cultured meat are arguably beginning to increase due to an increase in Scientific breakthroughs that are lowering the cost of production of cultured meat. This study aims to assess consumers' receptiveness to cultured meat by evaluating the valency of positive and negative perceptions toward cultured meat. It does this by reviewing the poll statistics of past studies that evaluated consumers' risk and benefit perceptions of cultured meat. The study's results indicated that consumers had a strong belief in the possible social, cultural and ethical benefits associated with cultured meat. The findings also showed that consumers' biggest concern was about its possible low quality. The regional analysis indicated that statistics from the North American countries showed very high positive perceptions about cultured meat while statistics from developing countries indicated the most reservations about the product. The results dispelled the belief that social and cultural issues would rank highest as hinderances to consumer acceptance in developing countries and highlighted differing motivators for acceptance in Eastern and Western Europe. The evidence-based knowledge reported in this paper is useful in giving insights of how a global cultured meat industry could be distributed and which perception areas would present the biggest challenges or drivers of consumer acceptance.

## Highlights

• The reviewed statistics showed that consumers generally had a strong belief in the possible social, cultural and ethical benefits that could associated with cultured meat.
• Statistics reported from North American consumer studies showed the highest optimism about cultured meat's introduction, while statistics from developing countries indicated the highest scepticism about cultured meat's potential benefits.
• Studies from Europe reported the greatest conflicts in opinions about cultured meat.

## 1 Introduction

Cultured meat represents one of the most recent controversial food technologies [1]. Proponents of cultured meat have lauded it as a product that would improve the current protein production systems as it is free of animal slaughter and, offers health and environmental advantages by reducing environmental pollution and resource use [2]. Its production also provides an opportunity for the sustainable development of designer, chemically safe, and disease-free meat [3]. On the other hand, cultured meat has been criticised for the potential economic, safety and health hazards that are associated with its production and consumption [4]. The debates among consumers, on whether cultured meat brings opportunities or threats to society have been carried out on various public platforms which include: social media cites, news and magazine outlets, and civil groups’ websites [[5], [6], [7]]. Academics have also shown an equal interest and have analysed the consumer psychology around cultured meat using both quantitative and qualitative methods (see [8]).

The increasing interest in consumers' future acceptance or rejection of cultured meat has resulted in fragmented views of consumer perceptions of this food technology (Bryant and Barnette, 2020; [4]). A common trend in this growing body of literature is the increasingly diverse areas of assessing consumer perceptions of cultured meat [[9], [10]]. While the variety in this literature offers helpful insights into the antecedents of consumer behaviour, the lack of uniformity in findings curtails one's ability to carry out comparisons between these studies' findings. This review aims to overcome this shortcoming by developing a thematic framework that can organize the previous studies' findings and facilitate comparisons across academic works. In addition to the challenge of non-uniform data, previous review studies have avoided the task of comparing the consumer perceptions across published academic studies because there exists a paucity of data on consumers' perceptions about cultured meat [11]. As a result, past studies such as Hartmann and Siegrist [12], Bryant and Barnett [9], Siddiqui et al. [13], Pakseresht et al. [4], Kouarfate and Durif [14] and Ye et al. [15] resorted to aggregating the review of consumers' perceptions with the review of the factors driving receptiveness, awareness and other similar aspects of cultured meat.

This study's principle contribution to literature is its measurement of the valency of the perceptions about cultured meat using data collected in a systematic review of past quantitative studies. This analysis' results provide insights into areas that could pose challenges or alternatively drive the global cultured meat industry's growth. Past efforts to review cultured meat's perception valency by Fernandes et al. [16] proved inconclusive with no consensus found on the perceived strengths, weaknesses, threats and opportunities of cultured meat. Empirical investigations have also attempted to provide an understanding of the perceived private and shared benefits/risks associated with cultured meat. These studies have indicated a growing consensus that personal risks are likely to outweigh the personal benefits of cultured meat, and a belief that social benefits are likely to accrue at the global level rather than at the individual level [[11], [17], [18], [19]]. However, substantive generalizations cannot be made from these studies due to their limited number, geographical spread, and scope of analysis. The current study overcomes these limitations by developing and using a five-theme framework to determine the key tenants of consumers' perceptions towards cultured meat and using it to analyse the statistics reported in past studies that have wider geographic spread. To its advantage, this analysis is guided by the social representative theory (Wagner and Kronberger, 2001), which states that consumer psychology should be analysed with respect to consumers' rationalisation of factors that represent their values, ideas and practices. Therefore, the study provides definitions of a variety of issues that are debated when positive and negative perceptions towards cultured meat are formed and provides a better understanding of consumers' psychology and better prediction of consumer behaviour. It also compares the results of evaluations of positive perceptions to negative perceptions of cultured meat and comments on the suitability of each approach in predicting consumers' behavioural antecedents. An additional strength of this study is its ability to conduct regional comparison of consumers' perception valency across studies from different parts of the world, which provides insights on the possible distribution of a global cultured meat industry.

## 2 Methods and materials

**Fig. 1:**
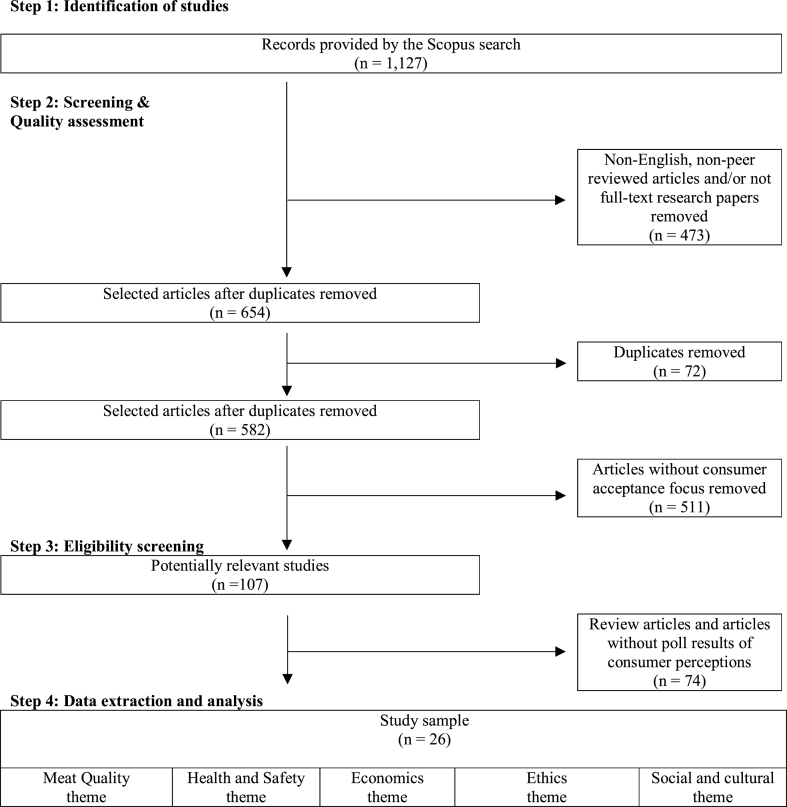
Flow diagram of systematic review map.

This study performed a systematic review of quantitative studies that reported the poll results of survey questions on consumers’ risk and benefit perceptions of cultured meat. The systematic review followed five steps (shown in Fig. 1) which involved identification and collection of studies, screening and quality assessment, eligibility assessment and data analysis.

### 2.1 Identification and collection of studies

Study identification and collection was done in two repetitive rounds. This was done to reduce the risk of leaving out studies as recommended by Stanley and Doucouliagos [20]. The first round of study collection was conducted between May and July 2023, while the second was done between December 2023 and January 2024. The literature search utilised the Google Scholar search engine of the University of South Africa and searched for journal articles published between 2013 and 2023. This selected period covers the time after cultured meat's first public tasting in London [15]. The research question for the study was, “What is the valency of consumers' perceptions toward cultured meat?” The search strategy included terms related to cultured meat and consumers' (positive and negative) perceptions as shown in Table 1. In place of the word “perceptions”’ words such as “attitudes”, “beliefs” and “emotions” were also used.

**Table 1:** Literature search strategy.

Search field		
#1	Cultured meat	“in vitro meat” OR “lab grown meat” OR “clean meat” OR “synthetic meat” OR “synthetic meat” OR “artificial meat” OR “animal-free meat” OR “test tube meat”
#2	Positive perceptions	“advantages” OR “gains” OR “prospects” OR “motivations”
#3	Negative perceptions	“risks” OR “disadvantages” OR “losses” OR “fear”
#4	#1 AND #2 #1 AND #3	

### 2.2 Quality screening and eligibility

As shown in Fig. 1, the internet search in Step 1 identified 1127 studies and in Step 2, this sample was subjected to the inclusion and exclusion criteria shown in Table 2. The first round of screening removed articles that were written in languages different from English. Quality screening was then done by removing non-full-text research papers and/or not published in peer-reviewed journals. After these two rounds of screening, the sample consisted of 654 studies. The sample was trimmed by removing 72 duplicate studies and 511 studies that lacked a consumer acceptance focus. A further eligibility test was conducted using Krippendorff's [21] approach for assessing content in Steps 3 and 4. This approach involved scanning through studies to identify text relevant to answering the specified research question. Hence, from the remaining 71 studies that discussed the cognitive analyses of consumer psychology towards cultured meat, the search drew out 26 articles that provided poll results of surveys indicating consumers' positive and negative sentiments towards cultured meat.

**Table 2:** Inclusion and exclusion criteria.

Inclusion criteria	Exclusion criteria
- English language - Full-text paper - Published in peer-reviewed journal - Presentation of the results of empirical studies - Quantitative studies - Studies reporting poll results of consumers' perceptions towards cultured meat	- Non-English written articles -Non-peer-reviewed sources (e.g. working papers) -Papers with no empirical data (e.g. review papers) -Focus outside consumer psychology -Qualitative studies - Studies failing to separate results on cultured meat from information on other faux meat -Studies not reporting poll results of consumers' perceptions towards cultured meat

### 2.3 Data extraction and analysis

Data for this study was derived from the statistics extracted from consumer perceptions about cultured meat reported in the 26 articles selected in Stage 4. As reporting on perception variables was not uniform in the literature (with each study reporting on a unique set of consumer perception indicators) the current study developed five thematic areas to categorise the perception indicators or statements that were reported. These thematic groups were developed from a scoping review that was conducted from the 74 studies in Step 3 and these defined the global themes on consumer perceptions about cultured meat. The Arksey and O'Malley's [22] approach to conducting a scoping review was utilised at this stage. It involved evaluating perception descriptions with respect to the context and the words used. Triangulation was used to develop working definitions of the thematic areas. The saturation point of a description of a thematic area was determined based on the thematic areas' ability to sufficiently capture the diverse but related aspects of the subject e.g. the meat quality theme comprised of perceptions cultured meat's possible texture, colour, smell, visual appeal and taste.

During data extraction, the perception indicators/statements from the collected studies were organised into the determined perception categories. Statements indicating a positive perception were separated from those indicating a negative perception and this was done because studies [[23], [24], [25]] have shown that consumers tend to respond differently when questioned about a negative aspect of a product than when they are questioned about a positive aspect.

Each perception statement/indicator was attached to a perception score allocated using a Likert scale by the authors of the articles selected for the current review. Using these statistics, three types of perception score averages were calculated. The first was the average perception score per study; where the mean of all the scores assigned to each perception statement in an article was calculated. Second was the average score per perception thematic category. In this metric, the mean of the perception statements classified in each of the five thematic areas developed in the current study was calculated. For the third metric, studies were classified into four geographic and economic regions (North America, Eastern Europe, Western Europe and Developing countries), and then an average perception score per region was calculated from the perception scores of countries falling in each of the four regions. These three metrics enabled the comparison of positive and negative perceptions across the five global themes and global regions.

Comparison of perceptions across thematic areas and regional groups was done using the averages weighted sample size of each theme/region with respect to the total population reported in the studies included in the current study. The average weighted perception per region/theme was calculated as shown in Eq (1) below:

(1) W_i_ = ∑X_i_*Y_i_

Where *W*_*i*_ represents the weighted average per thematic area or region, ∑ is a summation sign, *X*_*i*_ represents the sample proportion of a study *i*'s sample size with respect to the total sample of the theme/region measured and *Y*_*i*_ represents the perception score of the a given study's perception statement.

The studies selected for this review had perception scores measured on different (5-, 7- and 9-point) Likert scales. Hence, all data were rescaled to a 5-point Likert scale to make the statistics comparable. The 5-point scale was selected for this study due to its ease of interpretation and because the majority (77%) of the collected studies utilised this scale. The linear interpolation method was used to rescale the average scores of studies that measured consumers' perceptions. Interpolation enables the calculation of the value of one variable on a line within the range of an already established dataset [26]. The use of this method was made easy by the fact that all collected studies used odd-numbered Likert scales that had a mid-scale anchor point, which indicated a point of common consumers’ indifference (see Ref. [27]). The anchor point ensured the underlying meaning of the scores does not change and interpretation of the data is done in context and not rely solely on the numerical values. The linear transformation for rescaling the 7- an 9-point scales is shown in Eq (2) below.

(2) X_a_ = X_b_ (X_A_/X_B_)

Where *X*_*a*_ represents the calculated rescaled value on a 5-point Likert scale, *X*_*b*_ represents the perception score average calculated for a study or category, and equivalent to value, *X*_*A*_ represents the maximum value on the 7- or 9- point Likert scale, *X*_*B*_ is the maximum value on the 5-point Likert scale (5).

The current study did not collect common effect size data such as regression coefficients, correlations, standard errors, t-statistics or p-values. Hence, as recommended by Stanley and Doucouliagos [20], a scale was developed to determine the effect of size of the valency of consumers’ perceptions towards cultured meat. As shown in Table 3, perception average scores falling in the range of 0.5–1.5 were interpreted as indicating a very low valency (positive or negative perception), while the ranges 1.51–2.5, 2.51–3.5 and 3.51–4.5 were interpreted as low, high and very high valency, respectively.

**Table 3:** Effect size determination.

Score range	<0.5	0.5–1.5	1.51–2.5	2.51–3.5	3.51–4.5	>4.5
Valency interpretation	Very low (Rarely attained)	Very low	Low	High	Very high	Very low (Rarely attained)

The study conducted, three checks of publication bias. These checked for 1. Study dependence (reliance on one estimate/statistic, when studies report more than one or dependence on estimates are not strictly independent of each other), 2. Author dependence (reliance on studies done by the same author(s)) and 3. Spatial dependence (reliance on findings from authors that receive direct feedback from each other or are influenced by prior findings).

## 3 Results

### 3.1 Global themes around consumer perceptions about cultured meat

The scoping review determined that consumers perceptions congregated around five global themes. These were Meat quality, Health and safety, Economics, Ethics and, Social and Cultural themes. Table 4 provides a summary of extracts from the reviewed literature indicating the arguments promoting the negative and positive perceptions for each theme. As shown, the arguments about cultured meat's quality appear to be the most concise as they are summarised in a few descriptive statements. On the other hand, the arguments in the social and cultural theme are more diverse and cover a larger variety of issues. It can also be noted that there seems to be more arguments on the economic risks against cultured meat's introduction than those that highlight its benefits.

**Table 4:** Description of global thematic areas describing consumers’ perceptions toward cultured meat.

Themes	Benefits (Opportunities)	Risks (Threats)
**Economic theme**	**Cultured meat could improve consumers' economic wellbeing by:** - providing a lower priced protein source, which may result lower cost of living and improve food security [28,18].	**Cultured meat could threaten the economic wellbeing of:** - consumers by causing a market shift that will increase the price of conventional meat [29]. -consumers because it is not clear if it will provide value for money [30]. -farmers by causing a reduction in the demand for conventional meat [[3], [31]]. -farm workers because it could reduce the size of livestock industry and therefore reduce jobs [32].
**Quality theme**	**Cultured meat presents an opportunity to:** -gain better control over meat composition and quality by manipulating the flavour, fatty acid composition, fat content, and ratio of saturated to poly-unsaturated fatty acids through composition of the culture medium or coculturing with other cell types [[33], [34], [35]].	**Cultured meat:** -production process could result in compromises to the intrinsic attributes (visual appeal, taste, juiciness and texture) consumers have been provided by conventional meat [[10], [36]]. -does not embody the true definition of meat [37].
**Social and cultural theme**	**Cultured meat provides marketers the opportunity to:** -reduce the polar divide between meat-eaters and non-meat eaters [[23], [24], [25], [38]]. -free consumers of religious restrictions and increase the demand for certain meat types (Bhat et al., 2014). -sell to vegetarians that do not eat meat because of the ethical reasons associated with the conventional meat [18,39]. - allow a larger number of consumers the freedom to explore their food curiosity [37]. - cultured meat is interesting [40]. Positive perception are also indicated by consumer interest in cultured meat and described by the following statements. Cultured meat: -Is useful [41], -Is convinient [42], -Has potential [10]	**Conflicts with cultured meat are likely to emerge from:** -food culture: practices of eating resulting from reflections on culture, religious belief, identity and other factors as cultured meat is associated general perceived as infringements on personal agency [[28], [43], [44]]. - practical question about how to eat it, with who and how to cook it [30]. - the need for cultured meat in society [37]. **Fears are likely to arise due to contention inherent in the following trends:** -changes in the symbolic values associated with human society, e.g. relationships with nature or with animals [[11], [45]]. -society's increasing dependency on technology [46]. - society's growing estrangement or alienate from nature [39]. -a feeling of deception that exists around novel food [30]. -concern about the loss of traditional agricultural practices, the loss of cultural practices around meat consumption [11].
**Health and** **safety** **theme**	**Cultured meat is good for my health because:** - it does not use antibiotics [34,47]. - it circumvents many of the issues associated with conventional meat production systems, like nutrition-related diseases, foodborne illnesses, antibiotic-resistant pathogen strains [39]. - it is produced in a fully controlled environment and without any potential contamination from the digestive organs of “neighbouring animals” [48].	Some consumers perceive that the (artificial) chemical components in its culture medium or the biomaterials of that could be harmful to their health could have or have an inhibitory effect on the health benefits of some micronutrients such as iron [[48], [49], [50]].
**Ethics theme**	**The production of cultured meat may result in improvements in animal welfare because:** - it will reduce the excessively brutal slaughter of animals. -has the potential to greatly reduce animal suffering while satisfying all the nutritional and hedonic requirements of meat eaters [39]. **The production of cultured meat is viewed as resulting in positive environmental consequences because:** - it is associated with lower land and water use than current meat production systems [40]. -it is associated with lower carbon and ecological footprint of meat products [32]. . -it could result in a return of land to wilderness which may help in restoration of many endangered species [39].	**The morality of cultured meat production threatens the preservation of animal rights because:** -cultured meat's production uses of bovine serum as this involves ending the life of a calf [51]. -live animals will be regularly subjected to biopsies to provide stem cells [32].. A drastic reduction in the number of farm animals may not be without consequences for animal biodiversity. What will be the future of pastures, agriculture, landscapes, and the countryside with fewer farm animals? [32]..

### 3.2 Results of consumers’ perception valency analysis across global thematic areas

#### 3.2.1 Perceived benefits associated with cultured meat across global thematic areas

Twenty-three studies reported the polling results of their investigations on consumers' positive perceptions toward cultured meat. Most studies tended to evaluate consumers' health and safety, ethical and quality benefit perceptions while perceptions on the social and cultural compatibility of cultured meat received the least attention. As shown in Table 5, there were marked differences in the valency of the positive perceptions about cultured meat. This is indicated by the wide range of average scores of the positive perceptions measured in the studies, which ranged from a low of 2.16 [35] and a high of 3.63 [42]. Assessing the average valency per study according to the effect size criteria set in this review, one finds that the majority of studies (15 studies (56%)) reported average scores falling between 2.5 and 3.5; indicating a generally high positive perception about the benefits of cultured meat. Only 2 studies [35,52] reported study average scores that fell within the 1.5–2.5 range, which indicated very low positive benefit perceptions. Five studies (20%) had study average perception scores that were greater than 3.5, which indicated a very high benefit perception of cultured meat. When comparing the valency across themes, the study's results of the weighted average score per theme's calculation show that there is a general view that the introduction of cultured meat is likely to result in very high social and cultural benefits. The remaining four perception categories' global scores fell with the 2.5–3.5 range and indicated a general high benefit perception in these thematic areas. The results indicate marginally higher weighted ethical (average = 3.33), health and safety (average = 3.17) benefit perceptions when compared to the weighted economic (average = 2.93) and quality benefits (average = 2.78).

**Table 5:** Estimating the valency of consumers’ positive perceptions towards cultured meat across global thematic areas.

Authors	Country	Sample size	Group of interest	Quality	Health and safety	Economic	Ethical	Social and cultural	Average score per study
*Verberke et al. [49]	Belgium	180	Flanders	2,85	3,08	3,31	3,51	–	3188
Gomez-Luciano et al. [28]	UK	180	None	2,57	3,18	2,57	3,08	–	2850
	Spain	200	None	2,36	2,93	2,65	2,99		2733
	Brazil	216	None	2,41	3,04	2,4	3	–	2713
	Dominican Republic	133	None	2,65	3,03	2,86	2,81	–	2838
Mancinia and Antonioli [47]	Italy	525	None	2,83	3,19	3,36	3,56	–	3235
Bryant and Dillard [53]	USA	480	None	3,27	3,5	–	3,98	3,7	3613
Weinrich et al. [40]	Germany	713	None	2,51	2,96	3,1	3,55	–	3030
Dupont and Fiebelkorn [35]	Germany	718	Young people	1,72	–	–	2,59	–	2155
Verberke et al. [37]	Belgium	398	Flanders	–	2,37	3,19	3,29	2,93	2945
*Cornelissen [10]	Netherlands	288	None	2,89	2,74	–	3,24	3,49	3090
Faletar and Cerjak [34]	Croatia	411	None	2,97	2,94	2,78	3,23	–	2980
*Krings [54]	Canada	312	Omnivores	–	3,69	–	3,32	3,75	3587
*Leung et al. [55]	Singapore	948	None	3,16	3,29	–	3,46	3,35	3315
Baybars et al. [52]	Turkey	417	None	1,85	2,3	2,17	2,82	–	2285
Owokorinan et al. [41]	Nigeria	233	None	–	4	3,63	–	3,31	3647
*Chong et al. [56]	Singapore	948	None	–	3,29	3,31	–	3,35	3317
*Ankiel et al. [10]	Poland	418	None	–	3,6	3,83	–	3,45	3627
Dempsey and Bryant [57]	China	1020	None	3,58	3,32	–	3,26	–	3387
Morais-da-Silva et al. [42]	Brazil	35	Food experts	–	4	3,63	–	3,31	3647
	Europe	56		–	3,29	3,31	–	3,35	3317
	USA	45	None	–	3,6	3,83	–	3,45	3627
Quevedo-Silva et al. [58]	Brazil	304	None	3,58	3,32	–	3,26	–	3387
**Weighted average score per theme**				2,78	3,17	2,93	3,33	3,59	3,16

Note: Studies with asterisks were evaluated with 7- or 9-pointed Likert scale but were recalculated to base 5 to ensure comparability.

#### 3.2.2 Perceived risks associated with cultured meat across global thematic areas

Sixteen studies reported the poll results of their evaluations of consumers’ negative attitudes towards cultured meat. The findings of these studies are shown in Table 6. Most of these studies discussed the economic risks, ethical perceptions (especially unnaturalness) about cultured meat and health and safety perceptions (particularly disgust perceptions). As shown, the average study scores ranged from a low of 2.41 [53] to a high of 3.95 [42]. Assessing the average valency per study according to the effect size criteria set in this review, one finds that the majority of studies (11 studies (69%)) reported an average score lying between 2.5 and 3.5; indicating a generally high negative perception about the benefits of cultured meat. Only 2 studies [35,52] reported study averages that fell within the 1.5–2.5 range, which indicated very low risk perceptions. Three studies (19%) had study average perception scores that were greater than 3.5, which indicated a very high risk perception of cultured meat. However, all the weighted average score per theme metrics indicated that there is no significant difference in the perceptions across the themes. In all, results indicate that there is a general view that the introduction of cultured meat is likely to result in high risk, with marginally higher negative perceptions about the economic (average = 3.29) and the quality (average = 3.27) risks as compared to the ethical (average = 3.03) and health and safety risks (2.83).

**Table 6:** Estimating the valency of consumers’ negative perceptions towards cultured meat across global thematic areas.

Authors	Country	Sample size	Group of interest	Quality	Health and safety	Economic	Ethical	Social and cultural	Average score per study
*Verberke et al. (2015a)	Belgium	180	Flanders	2,85	3,08	2,37	3,51	3,27	3,02
Weinrich et al. [40]	Germany	713	None	–	–	3,67	3,2	–	3,44
Bryant and Dillard [53]	USA	480	None	2,38	2,15	2,7	2,4	–	2,41
Dupont and Fiebelkom [35]	Germany	718	Young people	–	2,41	–	1,9	3,06	2,46
Wilks et al. [59]	USA	904	None	3,56	3,11	–	–	–	3,34
Verberke et al. [37]	Belgium	398	Flanders	3,56	3,47	–	3,32	2,96	3,33
Faletar and Cerjak [34]	Croatia	411	None	–	1,66	3,17	2,63	2,82	2,57
Tsvakirai et al. [2]	South Africa	658	None	3,47	3,15	3,3	2,99	2,23	3,03
Owokoniran et al. [41]	Nigeria	233	None	–	3,16	3,32	3,39	3,28	3,29
*Chong et al. [56]	Singapore	948	None	3,69	3,76	3,72	3,66	–	3,71
Ankiel et al. [10]	Poland	418	None	3,4	2,3	3,6	2,25	3,7	3,05
Dempsey and Bryant [57]	China	1020	Food experts	–	–	2,65	4,06	3,69	3,47
Morais-da-Silva et al. [42]	Brazil	35	None	–	–	3,34	–	–	3,34
	Europe	56		–	–	3,43	–	–	3,43
	USA	45		–	–	3,95	–	–	3,95
Quevedo-Silva et al. [58]	Brazil	304	None	–	–	3,56	–	–	3,56

**Weighted average score per theme**				3,39	2,90	3,23	3,10	3,14	3,15

**Note:** Studies with asterisks were evaluated with 7- or 9-pointed Likert scale but were recalculated to base 5 to ensure comparability.

### 3.3 Results of valency analysis across multinational regions

#### 3.3.1 Perceived benefits associated with cultured meat across multinational regions

The results in Table 7 indicate that consumers' optimism towards cultured meat's benefits varies regionally. As shown, the statistics from samples drawn from North America indicated a very high benefit perception as indicated by the average regional perception score of 3.62. This score was markedly higher than those from the remaining three regions which fell in the 2.5–3.5 range. A regional average score of 2.94 was calculated from statistics reported from samples drawn in Western Europe, which was marginally higher than that of Eastern Europe score of 2.67 and marginally lower than the 3.24 score calculated for developing country samples. It can be noticed that the North American region had very high perception scores across four of its perception themes (except social and cultural benefit theme). These indicate that the reviewed studies reported that consumers in North America were very optimistic about the health, safety, economic, ethical, social and cultural benefits that would result from the introduction of cultured meat. The main perceived benefit reported for the North American region was economical in nature, while statistics from all other regions indicated the greatest optimism on the possible social and cultural benefits that would be associated with cultured meat. There was limited consensus on the second highest area of benefit across the regions. While the North American (average = 3.72), Western European and UK (average = 3.16) regions indicated that the second-best strength of cultured meat would probably be the ethical benefits that with will be realised, studies from the Eastern Europe (average = 3.16) and the developing countries (average = 3.34) indicated that consumers thought that economic benefits and health and safety benefits would be second-best. There was consensus from all regions that the quality benefits of cultured meat are going to be most probably the lowest of all the five benefit categories.

**Table 7:** Comparing the valency of consumers’ positive perceptions towards cultured meat across geographical and economic regions.

Region	Quality	Health and safety	Economic	Ethical	Social and cultural	Average score per region
**North America**	3,27^a^	3,57^a^	3,83^a^	3,72^a^	3,71	3.62 (0.216)
**Western Europe and the UK**	2,57	2,93	2,51	3,16	4,07^a^	2.94 (0.184)
**Eastern Europe**	2,77	2,91	3,16	2,84	3,17	2.67 (0.184)
**Developing countries**	3,08	3,34	2,77	3,41	3,59	3.24 (0.320)

**Note**: The regions were defined as follows: North America = USA and Canada; Western Europe and UK = Belgium, Netherlands, the United Kingdom, Germany, Spain and Italy; Eastern Europe = Croatia and Turkey; Developing countries = Singapore, Brazil, Nigeria, China and South Africa.

**Note.**

a Indicates the region with the highest perception score in a global theme.

#### 3.3.2 Perceived risks associated with cultured meat in multinational regions

Table 8 shows that the average scores of all the four regions fell in the 2.5–3.5 range indicating that there was a general high-risk perception that dominated consumers views of cultured meat regardless of their differences in economic standing and geographical locations. As indicated by the differences in standard deviations of the calculated average scores per region, there was more consensus in the perceptions reported by study participants drawn from developing countries, North America and Eastern Europe than those reported by participants drawn from Western Europe. Probing the perception category with the highest concern, the results show that studies from North America (average = 3.15) and Western Europe (average = 3.40) indicated that consumers were most sceptical about cultured meat's quality. The samples drawn from the developing countries and from Eastern Europe indicated that the consumers in these regions were most concerned about the ethical implications (average = 3.63) and on the economic implications (average = 3.41) of introducing cultured meat, respectively. Comparison across themes shows that the results from the studies carried out in the developing countries indicated the highest meat quality scepticism (average = 3.60), health and safety risk perception (average = 3.37) and ethical concern (3.63). The highest concern about the negative economic threats and social cultural implications associated with the introduction of cultured meat were reported by consumers from Eastern (average = 2.41) and Western (average = 3.26) Europe, respectively.

**Table 8:** Comparing the valency of consumers’ negative perceptions towards cultured meat across geographical and economic regions.

Region	Meat quality	Health and safety	Economic	Ethical	Social and cultural	Average score per region
**North America**	3,15	2,78	2,81	2,40	–	2,78 (0.307)
**Western Europe**	3,40	1,98	3,39	2,44	3,26^a^	2,89 (0.647)
**Eastern Europe**	3,34	2,83	3,41^a^	2,79	3,06	3,08 (0.284)
**Developing countries**	3,60^a^	3,47^a^	3,21	3,63^a^	3,14	3.41 (0.244)

**Note**: The regions were defined as follows: North America = USA; Western Europe = Belgium and Germany; Eastern Europe = Croatia and Poland; Developing countries = South Africa, Nigeria, Singapore, China, Brazil. There were no statistics reported for the UK.

**Note.**

a Indicates region with the highest perception score in a thematic areaStandard deviations of average regional scores are parenthesis.

## 4 Discussion

The scoping review conducted in this study showed that the research published during the 2013–2023 period largely investigated the quality, health and safety, economic, ethical and, social and cultural issues surrounding cultured meat's production, consumption and commercialisation. The scoping review found that the arguments in the meat quality and health and safety themes were concise and succinct while those in the social and cultural theme were diverse. The explanations for the economic risk associated with the introduction of cultured meat were also found to outnumber those offered to explain its possible benefits. The clarity in the definition of concerns around health, safety, economics and ethics issues surrounding cultured meat could contribute to the reason why these areas were among the most investigated as shown in the systematic review.

Although there was a larger data set for positive perceptions than negative perceptions towards cultured meat, both set of studies yielded similar findings on the valency of perception towards cultured meat. This indicates the appropriateness of using either positive or negative perceptions as estimates of the antecedents of consumers' future acceptance of cultured meat. As illustrated in Table 9, the review found that consumers are more optimistic that the introduction of cultured meat is likely to bring very high social and cultural benefits (low social and cultural risk) and very high meat quality compromises (very low quality improvements). Additionally, the results indicate that the risk and benefit expectation for the economic benefit/risk and ethical benefit/risk were commensurate with the same pattern which indicates a clarity in consumers’ opinion. However, inconsistencies in opinion can be noted in ranking of the health and safety issues and in social and cultural issues. As shown, a very positive perception on the social and cultural aspects of cultured meat was not matched with a low risk ranking, while a very low risk ranking on the health and safety aspects was not matched with a high ranking on the benefits of cultured meat. This inconsistency in results points to an area that could require additional research.

**Table 9:** Summary of global perceptions.

	Meat quality	Health & Safety	Economic	Ethical	Social & Cultural
Ranking on positive perceptions among thematic areas (Benefit perception)	**5**th (Very low)	**3**rd (Unsure)	**4**th (Low)	**2**nd (High)	**1**st (Very high)
Ranking on negative perceptions among thematic areas (Risk perception)	**1**st (Very high)	**5**th (Very Low)	**2**nd (High)	**4**th (Low)	**3**rd (Unsure)

When comparing these results with literature, these results are not entirely inline previous studies. The congruence in the conclusions provided by positive perceptions and the negative perception analyses is opposed to the findings by Sanchez-Sabate and Sabate [60] who advocate for the use of positive perception indicators as opposed to negative perception indicators. The current study's results only partially oppose this notion, therefore this could be an area for further consumer psychology research. Another controversial finding of this study is its elicitation that people had relatively low health and safety risk perceptions when compared with the other four areas of possible risk. This was an interesting finding as past studies have placed cultured meat's health and safety concerns among the highest concerns. The study enriches the literature on consumers' perceptions about cultured meat by aligning the research in this field with the social representative theory, which states that analysing the consumer psychology requires the rationalisation of factors that represent their values, ideas and practices (Wagner and Kronberger, 2001). It acknowledges that consumer perceptions are socially constructed and created from the debating of both positive and negative views of an unfamiliar subject.

The study's results on the multi-regional comparison highlighted the significantly high optimism reported in the studies in North America. This region had the highest benefit perception scores for most (4) cultured meat perception thematic areas and the lowest risk perception scores for three of the thematic areas. It was interesting to note that the developing countries' perception followed an almost opposite trend, where this region's risk perception scores for cultured meat's ranked high and its benefit perceptions were low. It was interesting to note that the studies from Europe (both East and West) and developed countries indicated that consumers perceived the social and cultural benefits as highest possible benefits that could be associated with cultured meat. The Western European region statistics indicated that consumers in region had a high expected economic and ethical benefit. The positive perceptions about the ethical and economic benefits found in Western Europe studies can be corroborated with findings reported by Klockner et al. [61]. Klockner et al. [61] focused on Western Europe countries (Denmark, Finland and Norway) and found that consumers were most convinced of the possible ethical and economic benefits associated with cultured meat as opposed to other health and safety benefits. The current study's results indicate that the optimism of the ethical and economic benefits of cultured meat is higher than the optimism of the quality, health and safety benefits in Europe and the UK. This finding is similar to results reported by Grasso et al. [62] who conducted a study in UK and Europe (Finland, Netherlands, Poland and Spain) and indicated that cultured meat acceptance would be motivated by economic (price), social (convenience), quality, ethical (sustainability) and health benefits, respectively. The current study showed high perceived ethical benefits in North America and Developing countries. Similar findings were also reported by Siegrist and Hartmann [63] who found high ethical acceptance (high naturalness) of cultured meat in USA, South Africa and Mexico when compared to China, France, Germany, England, Spain and Sweden.

The current study weighs in on the current debate (see Ref. [64,65]) about consumers in developing countries’ perceptions on the compatibility of cultured meat with their social and cultural institutions. It found that reports from this region were not too concerned about this as compared to other matters such as ethical, quality, health and safety implications of cultured meat. This result is contrary to Bekker et al. [45] and Gomez-Luciano et al. [28] who found that developing countries are likely to have higher negative perceptions (neophobia) towards cultured meat than developed countries. The results also disagree with the findings of Zhang et al. [66] who attributed the cultured meat neophobia in developing countries to relative higher negative experience with food regulation and poor health care system. However, the results agree with Ngah et al. [67] carried out a multi-national study on 12 African countries (Cameroon, Congo, Democratic Republic of Congo, Ghana, Ivory Coast, Kenya, Morocco, Nigeria, Senegal, South Africa, Tanzania and Tunisia) and found that quality concerns ranked fourth and that the biggest concerns African consumers congregated around health, safety and ethical issues and then followed by economic concerns.

## 5 Study limitations and future studies

This study is not without limitation. First, the sample of studies was small and not representative of the global population. Some countries were not represented and data in some categories was not provided in the study because the statistics were not available in literature. Second, the classification of countries into regions was applied using two overlapping criteria (geographical and economic criteria) and countries in regions were not proportionally representative of the global population distribution (i.e. there was an over representation of Western Europe). Lack of representation of other countries introduced some spatial bias in the findings and also limited the definitions of global themes describing consumer perceptions. A certain degree of author or study dependence was noted in the study as the results from some author consistently provided statistics that were significantly higher and lower than the those reported in other studies. The use of theme and regional averages during analysis, solved this problem.

As the number of studies included in this analysis was contingent on study availability and not on researchers' selection, it is recommended that future studies improve the representativeness of the sample and comparison between positive and negative perceptions of cultured meat. This representativeness will improve as more studies providing relevant data become available. Studies could also consider ways of classifying countries (e.g. disaggregating the developed countries’ and the Western European categories) or consumer perceptions (e.g. considering other categories). Future studies should also include meta-analysis of the factors affect consumer acceptance of cultured meat. These analyses have the advantage of collecting statistical effect sizes indicators such as p-values which improve the rigor of analysis performed during article review.

## 6 Conclusion

There has been a wide consensus that the understanding of consumer perceptions would be critical for driving the acceptance of cultured meat. This review identified the core domains of consumer perceptions. These were quality, health and safety, economic, ethical and socio-cultural perceptions. Using a framework constructed on these five core domains, this study evaluated the valency of consumers perceptions towards cultured meat. This evaluation of perception valency revealed the areas of influence in consumers' acceptance of cultured meat. Social and cultural benefits (minimal risks) were identified as strong drivers and were found to be stronger motivators than health and safety benefits (minimal risk) which were classified as relatively strong drivers. On the other hand, meat quality risks (minimal benefits) were identified as strong repulsions to cultured meat. The second most popular reason to cause possible rejection to cultured meat was ethical risk as this provided the second weakest encouragement for cultured meat's adoption. Perceived economic risks and benefits were not likely to sway the consumers towards or from cultured meat.

The use of a greater variety of perception domains also allowed for comparison studies. This was been missing in the literature. Interestingly, the study found that consumers from the Northern American region had very high positive perceptions about cultured meat. This finding bodes well with the United States of America's decision to be part of the first nations to allow commercial production of cultured meat. It was interesting to also note that the social and cultural concerns ranked high globally and not of central importance in the developing countries. The findings of the areas of concern and motivations in the different regions highlighted the different areas of focus for future research and marketing effort. The results also provide an indication of the potential footprint of the cultured meat market. As shown by the sporadic nature of the perceptions, the market is not likely to be geographically structured but will have certain regions with higher acceptance. If the cost of cultured meat is not taken into account, there is market potential as indicated by consumer benefit perceptions for cultured meat in different parts of the world. However, additional research is required to achieve more insights on the market.

## Data availability statement

Data is available upon request.

## Ethics statement

This study was reviewed and approved by the University of South Africa's School of Business Leadership Ethical Committee, with the approval number: 2023_SBL_AC_004_EX _0897.

## CRediT authorship contribution statement

**C.Z. Tsvakirai:** Writing – original draft, Methodology, Investigation, Formal analysis, Data curation, Conceptualization.

## Declaration of competing interest

The authors declare that they have no known competing financial interests or personal relationships that could have appeared to influence the work reported in this paper.
